# 792. Outbreak of Multidrug-resistant *Pseudomonas aeruginosa* in a Tertiary Healthcare System in Detroit

**DOI:** 10.1093/ofid/ofab466.988

**Published:** 2021-12-04

**Authors:** Kenisha J Evans, Angela Beatriz Cruz, Monica Meyer, Lavina Jabbo, Mara Cranis, Lori Cullen, Judy Moshos, Teena Chopra, Teena Chopra

**Affiliations:** 1 DETROIT MEDICAL CENTER, DETROIT, Michigan; 2 Detroit Medical Center - Wayne State University, Detroit, Michigan; 3 Wayne State University School of Medicine, Detroit, Michigan; 4 Department of Infection Control, Detroit Medical Center, Detroit, MI, USA, Detroit, Michigan; 5 Detroit Medical Center, Lakeshore, Ontario, Canada; 6 Detroit Medical Center, Wayne State University, Detroit, MI

## Abstract

**Background:**

*Pseudomonas aeruginosa* is one of the most common causes of healthcare-associated infections in critically ill patients and those with suboptimal immunity. However, the development of multidrug resistant *Pseudomonas aeruginosa* (MDR Pa) creates an even great disease burden and threat to both the hospital and local community health. The purpose of this study is to illustrate a descriptive analysis of a cluster of MDR *Pseudomonas,* during a local surge of SARS-CoV-2 (COVID 19) pandemic. The goal is to shed more light on the troublesome parallel during outbreaks, such as COVID-19 and consequential secondary outcomes.

**Methods:**

From November 2020 through February 2021, 16 patients exposed to the intensive care units of a tertiary healthcare system were infected or colonized with a multidrug-resistant strain of P. aeruginosa (Figure 1). Outbreak investigation was conducted via retrospective chart review of the first eight cases and prospective analysis of the latter eight cases. The isolates collected prospectively were analyzed for taxonomic identification, antimicrobial resistance profile, and phylogenetic analysis. Clinical characteristics of all patients were collected, and epidemiological investigation was carried out. MDR is defined as resistance to at least four classes of antibiotics: third-generation cephalosporins, fluoroquinolones, aminoglycosides, and carbapenems.

Figure 1. Epidemiological Curve of Cases of Multidrug-resistant Pseudomonas aeruginosa in the Detroit Medical Center from November 2020-February 2021

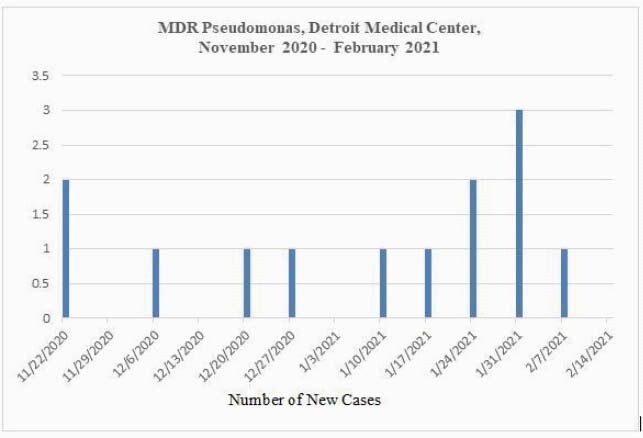

**Results:**

Of the 16 cases of MDR Pa infections, seven died within five months (Table 1). Antimicrobial resistance gene profiling detected blaOXA and blaPAO betalactamase genes in all the samples. One sample contained an additional blaVIM resistance gene, although this patient was colonized and not actively infected. The analysis suggests existence of two clusters demonstrating relatedness and possible horizontal transmission. Timing of this cluster of cases coincides with surge of COVID-19 cases. This highlights the importance of infection control measures and antimicrobial stewardship.

Table 1. Characteristics of patients infected with multidrug-resistant Pseudomonas aeruginosa (MDR-Pa) at Detroit Medical Center, November 2020 to February 2021

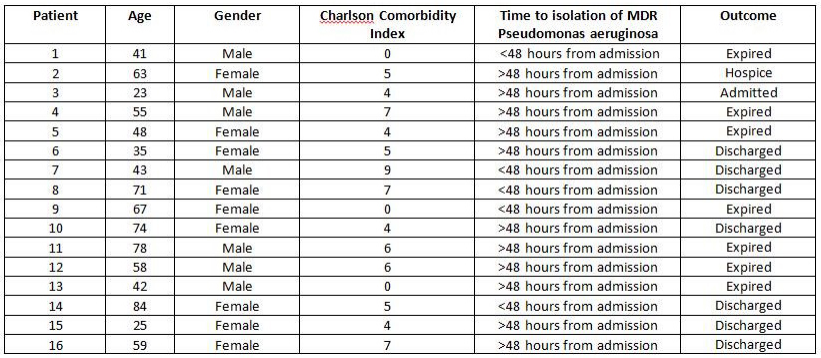

**Conclusion:**

Since early 2017 studies show there is a growing prevalence worldwide in transferable resistance, particularly for β-lactamases and carbapenemases, MDR Pseudomonas. This study emphasizes an irony paralleled during a pandemic, the needed efforts to prevent unintentional lapses in patient safety.

**Disclosures:**

**All Authors**: No reported disclosures

